# Geosmin Events Associated with *Dolichospermum circinale* Abundance Promoted by Nitrogen Supply in a Chinese Large Tropical Eutrophic Reservoir

**DOI:** 10.3390/microorganisms12122610

**Published:** 2024-12-17

**Authors:** Li-Juan Xiao, Yanru Jiang, Zihan Chen, Liang Peng, Yali Tang, Lamei Lei

**Affiliations:** 1 Department of Ecology and Institute of Hydrobiology, Jinan University, Guangzhou 510632, China; tljxiao@jnu.edu.cn (L.-J.X.); jiangyanru200223@163.com (Y.J.); czh10172022@163.com (Z.C.); tpengliang@jnu.edu.cn (L.P.); 2 Guangdong Engineering Research Center of Reservoir Cyanobacteria Bloom Control, Guangzhou 510632, China

**Keywords:** geosmin, *Dolichospermum circinale*, tropical, large reservoir

## Abstract

Taste and odor (T/O) compounds are a global threat in drinking water, mainly produced by cyanobacteria in freshwater environments. Temperature plays a crucial role in regulating geosmin dynamics in temperate and subtropical lakes, while its influence may be lower in tropical waters. To better understand the factors affecting geosmin occurrence in tropical waters, a dataset from a field investigation conducted in a large tropical reservoir was analyzed. The water temperature varied between 16 °C and 32 °C, with geosmin concentration ranging from below the detection limit (3 ng/L) to as high as 856 ng/L. Elevated geosmin levels exceeding > 10 ng/L were observed over the whole year except for in September, suggesting that the annual temperature was suitable for geosmin production. Among the diverse cyanobacteria, *Dolichospermum circinale* was identified as the main producer of geosmin in the reservoir, both by correlation analysis and cells’ geosmin measurements. Geosmin concentration was also significantly related to the abundance of *D. circinale.* None of the environmental variables (temperature, pH, transparency and nutrients) were significantly directly correlated with geosmin concentration. But the high total nitrogen significantly explained the increase in *D. circinale* abundance associated with geosmin elevation. Our results suggest that nutrients, particularly nitrogen, directly affected the competitive advantage and abundance of key geosmin producers and thus modified geosmin levels in this tropical reservoir. Our study thus hints at the possible management of the geosmin problem through nutrient reduction in tropical reservoirs.

## 1. Introduction

Geosmin, an earthy/musty compound, is one of the most important taste and odor (T/O) compounds affecting drinking water quality due to its low threshold concentrations and widespread presence in various water bodies which are resistant to standard water treatment processes [1,2,3,4,5,6]. Various benthic and pelagic microorganisms, including cyanobacteria and actinomycetes, can produce geosmin; thus, geosmin acts as a pollutant in water [7,8,9]. In the limnetic zones of lakes and reservoirs, cyanobacteria of the genera *Planktothrix*, *Dolichospermum* (formerly *Anabeana*), *Streptosporangium*, and *Symploca* are the main sources of geosmin [7,8,10,11,12]. To ensure the safety of drinking water supplies, it is increasingly important to accurately predict geosmin concentrations and implement effective treatments in water sources [13,14]. While the diversity of geosmin producers of different niches results in the varied responses of geosmin concentrations to environmental changes, it still is a challenge to predict geosmin events and control them through environmental management [1,13,15].

Geosmin synthesis is associated with the chlorophyll synthesis pathway and is regulated by several factors [13,16,17,18]. Among these factors, temperature is particularly important for the regulation of geosmin production at the cellular level [15,19,20,21]. The cellular expression of geosmin synthase (geoA) increased significantly under growth-inhibiting conditions at lower temperatures [7,13,19,22]. Experiments have also shown the optimal geosmin production and growth rate of *Anabaena* at 25 °C with a temperature scale of 10–35 °C [16,19]. Similar patterns were observed in culture experiments with *Lyngbya kuetzingii*. The algae showed a maximal geosmin concentration and productivity at 10 °C, with the highest chlorophyll a production at 25 °C [22]. Geosmin synthesis is sensitive to light intensity, as increasing light intensity favors less chl.a synthesis and more geosmin synthesis [16]. Moreover, cellular geosmin was promoted by ammonium-N, despite the fact that high nitrate-N levels suppressed geosmin production and promoted more chlorophyll a production [16]. Phosphorus serves an integral role in cellular metabolism and is required for all cyanobacteria and eukaryotic algae for growth. Not only biomass but also geosmin production is limited at low phosphate–phosphorus levels [16].

In natural waters, temperature is a crucial factor driving the seasonal dynamics of geosmin in temperate and subtropical regions. For example, in the Yuqiao Reservoir, a subtropical reservoir, the permanganate index and temperature were significant predictors of geosmin concentrations, with *D. circinale* and *Streptosporangium caverna* identified as the main producers [12]. Temperature significantly explained geosmin elevation in the subtropical Bukhan River during the summer [23]. Thus, climate change is expected to exacerbate geosmin events, as warming selectively benefits cyanobacteria, including geosmin producers [24,25,26]. According to a comparative study among nine reservoirs in Wales, UK, geosmin concentrations were significantly positively correlated with the ratios of inorganic nitrogen to phosphorus (TIN:TP) and ammonium to nitrate (NH_4_^+^:NO_3_^−^) [13]. A study at Plas Uchaf Reservoir (North Wales, UK) found that reducing phosphorus availability is crucial for the control of cyanobacterial growth and that concentrations of ammonium are key in the management of T/O outbreaks in drinking water reservoirs [27]. The results suggest that nutrient enrichment, especially ammonium enrichment, from anthropogenic activities in watersheds contributes to geosmin outbreaks in lakes [17,18,27]. In tropical waters, a year-round warm environment supported more diverse cyanobacteria, as well as geosmin producers. This complexity poses challenges to understanding and predicting the dynamics of geosmin based on temperature dynamics. As a matter of fact, the presence and prevalence of geosmin producers in local habitats are significantly constrained by resource availability. Typically, only a limited number of producers thrive in the limnetic zones of a specific lake or reservoir [28,29]. Thus, environmental management to control geosmin outbreaks should first assess the species pool of producers and identify the factors that trigger their blooming.

In the tropical zones of China, an increasing number of water supply reservoirs have faced threats from geosmin over the past 20 years (unpublished data), as the issue is aroused in lakes with warmer temperatures and in subtropical areas that consistently experience climate warming [30]. The phytoplankton assemblage in certain reservoirs is often dominated by just a few species, indicative of strong environmental selection pressures at play [31,32]. In these reservoirs, nutrients play a vital role in shaping the phytoplankton community, as well as in the dominance of cyanobacteria, regardless of the season in which cyanobacteria blooms and geosmin events are recorded [32,33,34]. More studies are needed to understand the role of environmental factors in the regulation of geosmin dynamics in warm waters.

Given the constant warm conditions and strong resource competition among phytoplankton species, as well as the importance of nitrogen in the regulation of geosmin synthesis, it is hypothesized that geosmin concentration depends on the abundance of a few key producers, and nutrients play a more important role than temperature in giving these producers a competitive advantage in warm waters, ultimately regulating the dynamics of geosmin. To test this hypothesis, we conducted a comprehensive analysis using data from an annual field investigation in a large tropical reservoir in Southern China, where a year-long geosmin event took place. This study contributes to our understanding of the mechanisms behind geosmin occurrence in warm waters.

## 2. Materials and Methods

### 2.1. Site Description and Sampling Time of the Dataset

**Figure 1:**
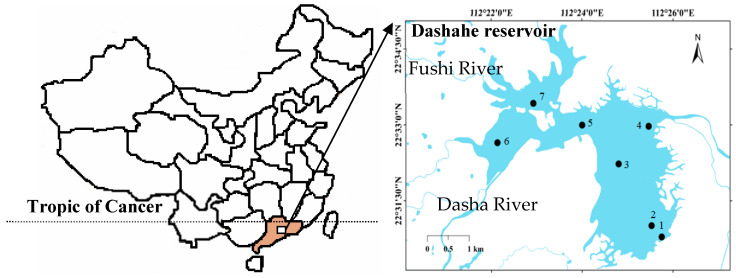
Location of Dashahe Reservoir in Guangdong Province (brown) and the sampling sites (No. 1–7) in the reservoir.

Dashahe Reservoir is a large tropical reservoir that supplies drinking water to a county with over a third of a million inhabitants [34]. Cyanobacteria blooms caused by *Microcystis* and *Dolichospermum* have occurred continuously since 2006, and the water emits a particularly strong musty/earthy odor all year round [33], so special attention has been paid to this troublesome odor problem. A seasonal investigation was conducted during March 2009 and February 2010 at 7 sampling sites across the reservoir (Figure 1). The two main rivers (Fushi River and Dasha River) feed into the reservoir near sampling site 6 and site 7, respectively, and the intake of the water treatment plant is located at site 1. The seven sites fall along a pronounced gradient of depth, from 2 m in the riverine zone to 14 m in the lacustrine zone.

### 2.2. Determination of Environmental Variables

At each sampling site, the water temperature (WT) and pH value were measured in situ with a Yellow Spring Instrument (YSI Inc., Yellow Springs, OH, USA). Water transparency (SD) was measured with a Secchi disk. For nutrient analysis, 500 mL of water was collected from a depth of 0.5 m below the water’s surface. Total nitrogen (TN), ammonia nitrogen (NH_4_-N), nitrate nitrogen (NO_3_-N), total phosphate (TP) and orthophosphate (SRP) were determined according to Chinese national standards for water quality [35]. Dissolved inorganic nitrogen (DIN) was calculated as the sum of the NH_4_-N and NO_3_-N concentrations. Another 200 mL of water was collected at a depth of 0.5 m in glass bottles and delivered under cooled conditions (4 °C in cooling boxes) for odor analysis.

### 2.3. Cyanobacteria Counting

For phytoplankton counting, one liter of water collected from 0.5 m below the water’s surface was fixed with Lugol’s iodine solution at a final concentration of 1% and allowed to sediment for 48 h prior to microscopic counting. All phytoplankton were identified at the species level by morphology [36,37]. The counting of cyanobacterial cell numbers for different species was performed using an inverted microscope [38], and biomass was computed using the biovolume method assuming a specific gravity of 1 mg/mm^3^ [39].

### 2.4. Measurement of Total Geosmin

Total geosmin was analyzed directly with the unfiltered water samples in this study. Geosmin in the water was analyzed using Headspace Solid-Phase Microextraction GC-MS (HSPME GC-MS), and geosmin standards were purchased from Supelco (Supelco, St. Louis, MO, USA). The method with a linearity range of 3–1000 ng/L was applied to raw surface water and was found to be highly automatized, reproducible and sensitive enough to detect the compound at a nanogram-per-liter concentration level.

### 2.5. Identification of Geosmin-Producing Species and Cells’ Geosmin Measurements

Potential geosmin producers in cyanobacteria were identified based on References [7,8]. The correlation between potential geosmin producers’ abundance and geosmin concentration was used to identify key producers. Then, we collected a sample in January 2010, and the filaments of key geosmin producers were microscopically picked using a glass capillary. After being washed several times with sterile distilled water, the filaments were subsequently cultured in a nitrogen-deleted BG11 medium for a couple of days. The raw culture was directly subjected to geosmin measurement (Table 1).

### 2.6. Statistical Analysis

The dataset included 10 environmental variables and all of the cyanobacterial taxa from the 84 samples. Multivariate regression analysis was performed to investigate the relationships of the response variable to multiple explanatory variables. Linear regression was used to identify the explanation of a single factor. The adjusted R-squared (R^2^_adj_) value and *p*-value in the F-statistic were used in the selection of significant variables. Significant cyanobacteria taxa and environmental variables were retained for variation partitioning analysis in order to test the interaction of the variables with geosmin concentrations. Spearman correlation analysis was used to detect the relationships between the environmental variables, and both linear regression analysis and the Mantel test were used to detect the explanatory effect of environmental variables on, mainly, producer abundance. All statistical analyses were performed in R platform version 4.0.0 [40,41].

## 3. Results

### 3.1. Environmental Conditions and Cyanobacteria Composition

**Figure 2:**
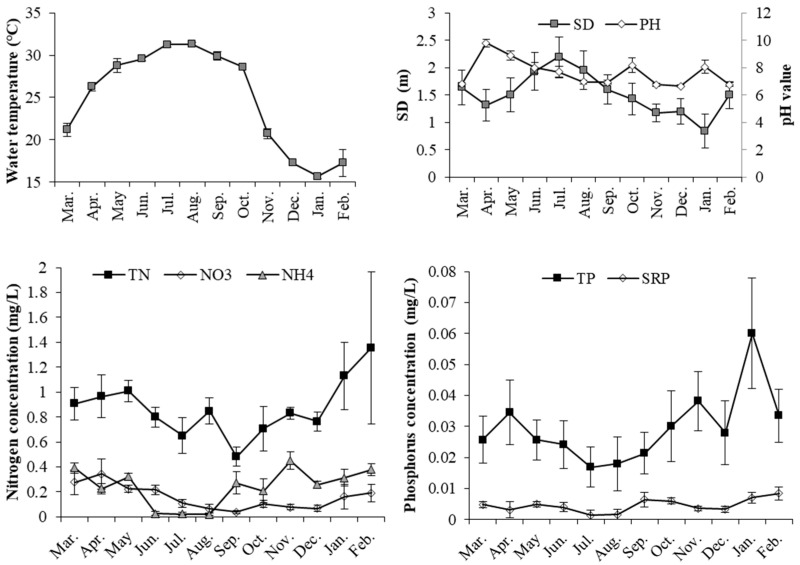
The seasonal dynamics of primarily environmental conditions including water temperature, transparency (SD), pH value, total and inorganic nitrogen and phosphorus concentration.

During the period of study, water temperature ranged from 16 to 32 °C in the studied reservoir. The light and total nitrogen and phosphorus conditions indicate a eutrophic state: an SD of 1.6 ± 0.46 m, a TN of 0.83 ± 0.22 mg/L and a TP of (0.028 ± 0.013) mg/L. The pH value was 7.2 ± 2.8, as a result of eutrophication. The SRP was 0.004 ± 0.002 mg/L, indicating a permanent phosphorus-limiting environment. The NH_4_-N was 0.23 ± 0.16 mg/L and the NO_3_-N was 0.15 ± 0.11 mg/L, indicating that the DIN of 0.38 ± 0.2 mg/L was mostly composed of NH_4_-N. The nitrogen and phosphorus supply increased after August through to January following a temperature decrease and were accompanied with a transparency decrease. This may indicate the seasonal relief of nutrient stress (Figure 2).

### 3.2. The Dynamics of Geosmin Concentration

**Figure 3:**
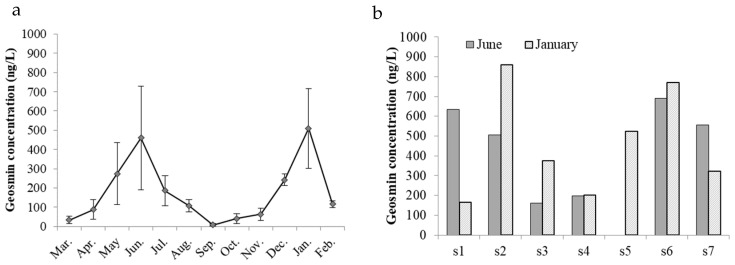
There were seasonal dynamics accompanied by an increase in spatial difference at two peak values of geosmin concentration in June 2009 and January 2020 (**a**), and a higher concentration was observed in both the deeper lacustrine (s1 and s2) and shallower riverine zones (s6 and s7) (**b**) in Dashahe reservoir. (s1 to s7 were the sampling sites No. 1–7).

The highest geosmin concentration was 859 ng/L, and the average concentration was 168 ± 187 ng/L. The annual concentration was above 10 ng/L except for in September, indicating an all-year-round geosmin event. Clear seasonality was observed, with two peak values in June, of 457 ± 225 ng/L, and in January, of 460 ± 269 ng/L (Figure 3a). Greater spatial heterogeneity was observed in the two months with peak values, and a higher concentration of >500 ng/L was found in both the deeper lacustrine zone (s1 and s2) and the shallower riverine zone (s6 and s7), which indicated the independence of geosmin concentration and depth (Figure 3b).

### 3.3. The Factors Affecting Geosmin Dynamics

**Figure 4:**
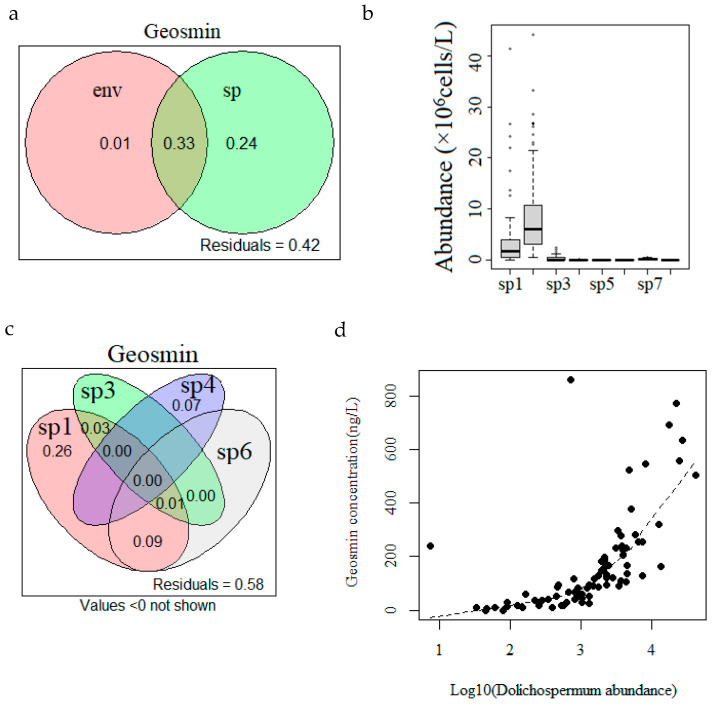
Variation partitioning analysis showed that cyanobacteria assemblage (labeled by sp), not environmental factors (labeled by env), significantly explained geosmin variance (**a**); eight cyanobacteria genera constituted the cyanobacteria assemblage (**b**); four cyanobacteria genera significantly related to geosmin concentration and *D. circinalis* abundance were the essential explanatory factors (**c**). The results of geosmin measurement of *D. circinalis* filaments confirmed that the increase in cell abundance caused the elevation in geosmin concentration (**d**). The labels of the eight cyanobacteria genera are as follows: sp1—*Dolichospermum*; sp2—*Microcystis*; sp3—*Pseudanabaena*; sp4—*Limnothrix*; sp5—*Raphidiopsis*; sp6—*Planktothrix*; sp7—*Dactylococcopsis*; sp8—*Planktolyngbya*.

No significant correlation was detected between environmental variables and geosmin concentration. The cyanobacteria assemblage was the mainly explanatory factor for the seasonal dynamics of geosmin (Figure 4a). The cyanobacteria assemblage included eight cyanobacteria genera: *Dolichospermum*, *Microcystis*, *Pseudanabaena*, *Limnothrix*, *Raphidiopsis*, *Planktothrix*, *Dactylococcopsis* and *Planktolyngbya* (Figure 4b). The dominant cyanobacterial species were *M. aeruginosa* and *D. circinalis.* For the cyanobacteria, significant positive regression relationships were detected between geosmin concentration and the abundance of four genera, including *Dolichospermum*, *Pseudanabaena*, *Limnothrix* and *Planktothrix* (Figure 4c). *D. Circinalis,* the only detected *Dolichospermum* species, was of average abundance (3.9 ± 6.9 × 10^6^ cells/L) and was the most important explanatory factor for geosmin concentration variance. The filaments of *D. circinalis* were microscopically picked out, and the geosmin concentration of the filaments was analyzed. This confirmed that the increase in *D. circinalis* abundance led to the significant increase in geosmin concentration in the water (Figure 4d).

### 3.4. The Driving Factors of the Seasonal Dynamics of D. circinalis Abundance

**Figure 5:**
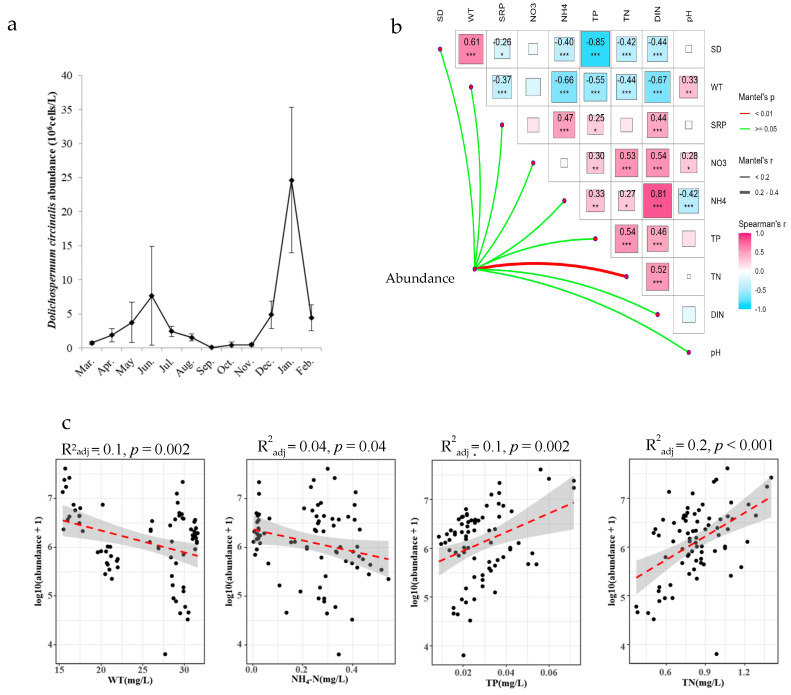
The seasonality of *D. circinalis* abundance (**a**); Spearman’s correlation analysis showed the strong collinearity among environmental variables, while only TN was significantly correlated with *D. circinalis* abundance based on the results of the Mantel test ((**b**), *: *p* < 0.05; **: *p* < 0.01; ***: *p* < 0.001); and the linear regression results of significant factors explaining *D. circinalis* abundance ((**c**), red dotted line was the fitting line, and the grey area is the double error range of fitting line).

The abundance of *D. circinalis* ranged from 0.06 × 10^6^ cells/L to 41 × 10^6^ cells/L (Figure 5a). In the Mantel test, only TN was a significant explanatory factor of the nine environmental factors (Figure 5b). Multiple stepwise regression analysis showed that WT, TN and NH_4_-N concentrations were significant explanatory factors for the dynamics of *D. circinalis* abundance during the period (*p* < 0.01, R^2^_adj_ = 0.55, df = 68). Of them, WT and NH_4_-N concentrations were negatively correlated and TN concentration was positively correlated with *D. circinalis* abundance. TP was significantly collinear with TN concentration; thus, the factor of TP was excluded from the multiple stepwise regression analysis as TN was more important in the analysis, even if TP concentration significantly positively explained *D. circinalis* abundance in the single-factor linear regression analysis (*p* < 0.01, R^2^_adj_ = 0.1, df = 74). The regression relationship between abundance and TN concentration was log10 (abundance + 1) =1.76 + 0.23 TN (*p* < 0.01, R^2^_adj_ = 0.2, df = 74), indicating that TN was the most important factor in the variance of *D. circinalis* abundance (Figure 5b).

## 4. Discussion

Our data from an annual field investigation in Dashahe Reservoir showed that geosmin concentration, averaging 168 ± 187 ng/L, exceeded 10 ng/L for the whole year, indicating an annual odor problem in this large tropical reservoir in Southern China. As previously reported in temperate and subtropical regions, temperature is a crucial factor driving the seasonal dynamics of geosmin in natural waters [12,23,24,25,26]. However, in our reservoir, temperature stayed high all year round and the annual temperature was suitable for geosmin production, as indicated by geosmin concentration data, suggesting a different geosmin outbreak model that gives less relevance to temperature.

The statistical analysis of field data and raw cells’ geosmin measurements both suggest that *D. circinalis* was the key geosmin producer in the reservoir. We focused our analysis on the relationship between geosmin concentration and cyanobacterial abundance, because numerous studies have confirmed that cyanobacteria are the major producers of geosmin in pelagic habitats [6,42] and other settings. Eight cyanobacteria genera were identified in our study, with four (*Dolichospermum*, *Pseudanabaena*, *Limnothrix* and *Planktothrix*) showing a significant relationship with geosmin concentration. *Planktothrix* and *Pseudanabaena* are known to produce 2-MIB, but only *Dolichospermum* has been confirmed to produce geosmin [7,43,44]. Previous studies have linked *Dolichospermum* (formerly *Anabaena*) to geosmin occurrence in field experiments of water bodies, such as in rivers and reservoirs in Australia, America and North Asia [45,46,47,48,49,50]. Laboratory cultures have also confirmed a positive relationship between *Dolichospermum* abundance and geosmin concentration under both pressurized and optimal conditions [45,51,52]. In this study, *D. circinalis* were the only *Dolichospermum* species detected.

The relationship between *D. circinalis* abundance and geosmin concentration is reliable in explaining odor dynamics. *D. circinalis* is a common blooming cyanobacterium and a significant contributor to geosmin production in natural waters worldwide [8,30]. Although temperature is important for predicting geosmin producer abundance in temperate lakes [13,26,53], the high abundance (>10^6^ cells/L) throughout the year confirms that temperature was not a limiting factor for *D. circinalis* occurrence in the warm tropical reservoir, where annual temperatures exceed 16 °C. The temperature range is suitable for *Dolichospermum* growth and allows blooms throughout the year [16,19], which also suggests that more attention should be paid to other factors regulating the abundance of *D. circinalis*.

Nitrogen was the most important factor explaining the seasonal dynamics of *D. circinalis* in this study. This was suggested by their positive regression relationships, confirming that eutrophication from increased nitrogen levels promoted its growth in the reservoir. Eutrophication is the primary cause of *D. circinalis* thriving and blooming in the studied reservoir and in other lakes [33,54]. In the reservoir, *Dolichospermum* coexisted with *Microcystis*, which both favor eutrophic water. Phosphorus is essential for phytoplankton growth; however, the relatively stable TP levels and low phosphorus availability indicated by the SRP concentration suggest a persistent eutrophic P-limiting reservoir. *Dolichospermum* has diverse strategies for adapting to P-limiting environments, including phosphorus storage mechanisms and organophosphorus utilization through extracellular alkaline phosphatase, contributing to their dominance in reservoirs with low phosphorus availability [54]. As for nitrogen, it was identified that the development of livestock in the basin contributed to the high total nitrogen and ammonium concentrations [55]. Experiments have confirmed that adequate ammonium supply is important for geosmin production [16,56], while ammonium is used easily in filaments and a negative correlation between geosmin concentration and ammonium frequently occurs in lakes [13,57]. In our study, the increased TN and even the stable DIN indicated an increased supply and utilization of nitrogen by *D. circinalis*. The higher concentration of NH_4_-N than of NO_3_-N indicated that ammonium primarily contributed to the nitrogen supply.

Our statistical analysis showed that neither temperature and light nor nutrients significantly explained the seasonal dynamics of geosmin concentration. Geosmin production in cells involves the MEP (2-methylerythritol-4-phosphate) and MVP (mevalonate) pathways, associated with the chlorophyll synthesis pathway [7,8,19]. It has been reported that temperature, light and nutrients fundamentally affect geosmin production by modifying its cellular synthesis [26,27,56,58]. However, the geosmin production capacity of *D. circinalis* is highly variable in the same nutrient and temperature conditions [19,22]. Here, although similar peak values of geosmin concentration were observed in both June and January, the abundance of *D. circinalis* in June was an order of magnitude lower than in January, suggesting a difference in the cellular geosmin production of *D. circinalis*. The more nutrient-stressed environment may have contributed to the higher geosmin production capacity in *D. circinalis* cells in June, with higher temperatures and light prevalence in the reservoir. On the contrary, the highest nitrogen and phosphorus supply and the highest abundance was in January, even though temperature and transparency were at the lowest levels. It was reported that a lower geosmin concentration occurred at a higher growth rate promoted by nutrient supply, even in low light conditions [19,20]. The variation in nutrient stress contributed to the change in the capacity of cellular geosmin production, which raised the uncertainty of the relationship between geosmin concentration and environmental factors. The results were consistent with our hypothesis: geosmin concentration depends on the abundance of a few key producers, and nutrients play a more important role than temperature in giving these producers a competitive advantage, ultimately regulating the dynamics of geosmin.

For the resolution of the geosmin problem, as implicated in our research, a nutrient reduction plan was recommended. The nutrients from agricultural production and domestic sewage were estimated with the Soil and Water Assessment Tool (SWAT) [55]. It was suggested that both the implementation of buffer zones along rivers and the removal of sewage discharges resulted in a marked improvement in reservoir water quality [55]. After ten years of management, there was a pronounced decrease in nitrogen concentration, while phosphorus levels more or less stayed the same [32,33]. As expected, *D. circinalis* numbers declined, and no geosmin events occurred anymore ([32,33] and unpublished data).

In aquatic ecosystems, some biological factors may also affect geosmin levels on small scales [9]. For example, biofilm formation may support geosmin action at microscale levels [59]. When producer cells are grazed by zooplankton, they were more likely to release geosmin [60]. Several bacteria strains capable of geosmin biodegradation were isolated [61,62]. Beyond nutrient control, more comprehensive resolutions, including degradative bacteria, biotic interactions (e.g., predators or competition) and biofilms, may need to be applied to control geosmin levels.

## 5. Conclusions

By a comprehensive analysis using data from an annual field investigation in a large tropical reservoir in Southern China, we found an annual odor event, with geosmin concentrations exceeding 10 ng/L. *D. circinalis* was the primary producer of geosmin, and its abundance showed a significantly positive correlation with geosmin concentration. Nitrogen was the critical factor modifying the *D. circinalis* population. The results suggest that in warm waters, nutrients, especially nitrogen, play a more important role than temperature in giving producers a competitive advantage and ultimately regulating the dynamics of geosmin. Our study thus hints at the possible management of the geosmin problem through nutrient reduction in tropical reservoirs, and more research is needed to confirm the response of geosmin to nutrient reduction in warm conditions.

## Figures and Tables

**Table 1:** Analytical conditions for geosmin.

Instruments	Conditions
GC-MS: 6890N-5975c (Agilent, Santa Clara, CA, USA)	Volume vial/volume liquid: 20 mL/10 mL
Detector: 5975c (Agilent, USA)	Extraction time: 25 min
Column: HP5-MS (30 m × 0.25 mm) (Agilent, USA)	Extraction temperature: 60 °C
Fiber HSPME: Stable Flex 50/30 μm DVB/CAR/PDMS (Supelco, USA)	Desorption time: 2 min
	Carrier gas: He 30 mL min^−1^
	Injection temperature: 250 °C
	Oven temperature: 40 °C (2 min); 40–250 °C (15 °C/min)
	Ionizer temperature: 230 °C
	Ionization voltage: 70 eV

GC-MS: gas chromatography–mass spectrometry; He: helium.

## Data Availability

The data that support the findings of this study are available from the corresponding author upon reasonable request due to ethical reasons.

## References

[1] Watson S.B., Ridal J., Boyer G.L. (2008). Taste and odour and cyanobacterial toxins: Impairment, prediction, and management in the Great Lakes. Can. J. Fish. Aquat. Sci..

[2] Howgate P. (2004). Tainting of farmed fish by geosmin and 2-methyl-iso-borneol: A review of sensory aspects and of uptake/depuration. Aquaculture.

[3] Jung S.W., Baek K.H., Yu M.J. (2004). Treatment of taste and odor material by oxidation and adsorption. Water Sci. Technol..

[4] Parinet J., Rodriguez M.J., Sérodes J. (2010). Influence of water quality on the presence of off-flavour compounds (geosmin and 2-methylisoborneol). Water Res..

[5] Dong Z.Y., Lin Y.L., Zhang T.Y., Hu C.Y., Pan Y., Zheng Z.X., Tang Y.L., Gao N.Y. (2021). The formation, analysis, and control of chlor (am) ination-derived odor problems: A review. Water Res..

[6] Mustapha S., Tijani J.O., Ndamitso M.M., Abdulkareem A.S., Shuaib D.T., Mohammed A.K. (2021). A critical review on geosmin and 2-methylisoborneol in water: Sources, effects, detection, and removal techniques. Environ. Monit. Assess..

[7] Jüttner F., Watson S.B. (2007). Biochemical and ecological control of geosmin and 2-methylisoborneol in source waters. Appl. Environ. Microbiol..

[8] Devi A., Chiu Y.T., Hsueh H.T., Lin T.F. (2021). Quantitative PCR based detection system for cyanobacterial geosmin/2-methylisoborneol (2-MIB) events in drinking water sources: Current status and challenges. Water Res..

[9] Garbeva P., Avalos M., Ulanova D., van Wezel G.P., Dickschat J.S. (2023). Volatile sensation: The chemical ecology of the earthy odorant geosmin. Environ. Microbiol..

[10] Su M., Gaget V., Giglio S., Burch M., An W., Yang M. (2013). Establishment of quantitative PCR methods for the quantification of geosmin-producing potential and *Anabaena* sp. in freshwater systems. Water Res..

[11] Wu A., Wang Y., Friese K., Zhang L., Han C., Kang D., Shen Q. (2021). Spatial and Seasonal Distribution of 2-Methylisoborneol in a Large Eutrophic Shallow Lake, China. Water Air Soil Pollut..

[12] Qiu P., Zhang Y., Mi W., Song G., Bi Y. (2023). Producers and drivers of odor compounds in a large drinking-water source. Front. Ecol. Evol..

[13] Hooper A.S., Kille P., Watson S.E., Christofides S.R., Perkins R.G. (2023). The importance of nutrient ratios in determining elevations in geosmin synthase (geoA) and 2-MIB cyclase (mic) resulting in taste and odour events. Water Res..

[14] Wang Y., Shao L., Kang X., Zhang H., Lü F., He P. (2023). A critical review on odor measurement and prediction. J. Environ. Manag..

[15] Cao J., Wu Y., Li Z.K., Hou Z.Y., Wu T.H., Chu Z.S., Zheng B., Yang P., Yang Y., Li C. (2024). Dependence of evolution of Cyanobacteria superiority on temperature and nutrient use efficiency in a meso-eutrophic plateau lake. Sci. Total Environ..

[16] Saadoun I.M.K., Schrader K.K., Blevins W.T. (2001). Environmental and nutritional factors affecting geosmin synthesis by *Anabaena* sp. Water Res..

[17] Jähnichen S., Jäschke K., Wieland F., Packroff G., Benndorf J. (2011). Spatio-temporal distribution of cell-bound and dissolved geosmin in Wahnbach Reservoir: Causes and potential odour nuisances in raw water. Water Res..

[18] Olsen B.K., Chislock M.F., Rebelein A., Wilson A.E. (2017). Nutrient enrichment and vertical mixing mediate 2-methylisoborneol and geosmin concentrations in a drinking water reservoir. Water Supply.

[19] Wang Z., Li R. (2015). Effects of light and temperature on the odor production of 2-methylisoborneol-producing *Pseudanabaena* sp. and geosmin-producing *Anabaena ucrainica* (cyanobacteria). Biochem. Syst. Ecol..

[20] Zhang T., Li L., Zheng L., Song L. (2017). Effects of nutritional factors on the geosmin production of *Lyngbya kuetzingii* UTEX 1547 (Oscillatoriales, Cyanobacteria). Phycologia.

[21] Clercin N.A., Druschel G.K. (2019). Influence of environmental factors on the production of MIB and geosmin metabolites by bacteria in a eutrophic reservoir. Water Resour. Res..

[22] Zhang T., Li L., Song L.R., Chen W. (2009). Effects of temperature and light on the growth and geosmin production of *Lyngbya kuetzingii* (Cyanophyta). J. Appl. Phycol..

[23] Hwan J.B., Kim H.N., Kang T.G., Kim B.H., Byeon M.S. (2023). Study of the cause of the generation of odor compounds (geosmin and 2-methylisoborneol) in the Han River system, the drinking water source, Republic of Korea. Water Supply.

[24] Taranu Z.E., Gregory-Eaves I., Leavitt P.R., Bunting L., Buchaca T., Catalan J., Domaizon I., Guilizzoni P., Lami A., McGowan S. (2015). Acceleration of cyanobacterial dominance in north temperate-subarctic lakes during the Anthropocene. Ecol. Lett..

[25] Kakouei K., Kraemer B.M., Anneville O., Carvalho L., Feuchtmayr H., Graham J.L., Higgins S., Pomati F., Rudstam L.G., Stockwell J.D. (2021). Phytoplankton and cyanobacteria abundances in mid-21st century lakes depend strongly on future land use and climate projections. Glob. Change Biol..

[26] Shen Q., Wang Q., Miao H., Shimada M., Utsumi M., Lei Z., Zhang Z., Nishimura O., Asada Y., Fujimoto N. (2022). Kazuya Shimizu Temperature affects growth, geosmin/2-methylisoborneol production, and gene expression in two cyanobacterial species. Environ. Sci. Pollut. Res..

[27] Perkins R.G., Slavin E.I., Andrade T.M.C., Blenkinsopp C., Pearson P., Froggatt T., Godwin G., Parslow J., Hurley S., Luckwell R. (2019). Managing taste and odour metabolite production in drinking water reservoirs: The importance of ammonium as a key nutrient trigger. J. Environ. Manag..

[28] Sommer U., Adrian R., De Senerpont Domis L., Elser J.J., Gaedke U., Ibelings B., Jeppesen E., Lürling M., Molinero J.C., Mooij W.M. (2012). Beyond the Plankton Ecology Group (PEG) model: Mechanisms driving plankton succession. Annu. Rev. Ecol. Evol. Syst..

[29] Huo D., Gan N., Geng R., Cao Q., Song L., Yu G., Li R. (2021). Cyanobacterial blooms in China: Diversity, distribution, and cyanotoxins. Harmful Algae.

[30] Harris T.D., Reinl K.L., Azarderakhsh M., Berger S.A., Berman M.C., Bizic M., Bhattacharyaf R., Burnetg S.H., Cianci-Gaskillh J.A., De Senerpont Domis L. (2024). What makes a cyanobacterial bloom disappear? A review of the abiotic and biotic cyanobacterial bloom loss factors. Harmful Algae.

[31] Lei L.M., Peng L., Huang X.H., Han B.P. (2014). Occurrence and dominance of *Raphidiopsis raciborskii* and dissolved cylindrospermopsin in urban reservoirs used for drinking water supply, South China. Environ. Monit. Assess..

[32] Xiao L.J., Lei L.M., Peng L., Lin Q.Q., Naselli-Flores L. (2021). Iron operates as an important factor promoting year-round diazotrophic cyanobacteria blooms in eutrophic reservoirs in the tropics. Ecol. Indic..

[33] Xiao L.J., Han B.P., Lin Q.Q., Lei L.M. (2007). Usage of flocculation in emergent control of algal bloom in drinking water supplying reservoir. Environ. Sci..

[34] Xiao L.J., Xie J., Tan L., Lei L.M., Peng L., Wang Z., Naselli-Flores L. (2022). Iron enrichment from hypoxic hypolimnion supports the blooming of *Raphidiopsis raciborskii* in a tropical reservoir. Water Res..

[35] American Public Health Association (1989). Standard Methods for the Examination of Water and Wastewater.

[36] Guiry M.D., Guiry G.M. (2024). AlgaeBase. World-Wide Electronic Publication. University of Galway. https://www.algaebase.org.

[37] Hu H., Wei Y. (2006). The Freshwater Algae of China: Systematics, Taxonomy and Ecology.

[38] Utermöhl H. (1958). Zur Vervollkommnung der quantitativen Phytoplankton-Methodik. Mitteilungen Int. Ver. Theor. Und Angew. Limnol..

[39] Hillebrand H., Dürselen C.D., Kirschtel D., Pollingher U., Zohary T. (1999). Biovolume calculation for pelagic and benthic microalgae. J. Phycol..

[40] Borcard D., Gillet F., Legendre P. (2011). Numerical Ecology with R.

[41] R Core Team (2023). R: A Language and Environment for Statistical Computing.

[42] Asquith E., Evans C., Dunstan R.H., Geary P., Cole B. (2018). Distribution, abundance and activity of geosmin and 2-methylisoborneol-producing Streptomyces in drinking water reservoirs. Water Res..

[43] Smith J.L., Boyer G.L., Zimba P.V. (2008). A review of cyanobactarial odorous and bioactive metabolites: Impacts and management alternatives in aquaculture. Aquaclture.

[44] Cao T., Fang J., Jia Z., Zhu Y., Su M., Zhang Q., Song Y., Yu J., Yang M. (2023). Early warning of MIB episode based on gene abundance and expression in drinking water reservoirs. Water Res..

[45] Bowmer K.H., Padovan A., Oliver R.L., Korth W., Ganf G.G. (1992). Physiology of geosmin production by *Anabaena circinalis* isolated from the Murrumbidgee River, Australia. Water Sci. Technol..

[46] Jones G.J., Korth W. (1995). In situ production of volatile odour compounds by river and reservoir phytoplankton populations in Australia. Water Sci. Technol..

[47] Smith V.H., Sieber-Denlinger J., deNoyelles F., Campell S., Pan S., Randtke S.J., Blain G., Strasser V.A. (2002). Managing taste and odor problems in a eutrophic drinking water reservoir. Lake Reserv. Manag..

[48] Uwins H.K., Teasdale P., Stratton H. (2007). A case study investigating the occurrence of geosmin and 2-methylisoborneol (MIB) in the surface waters of the Hinze Dam, Gold Coast, Australia. Water Sci. Technol..

[49] Byun J.H., Hwang S.J., Kim B.H., Park J.R., Lee J.K., Lim B.J. (2015). Relationship between a dense population of cyanobacteria and odorous compounds in the North Han River system in 2014 and 2015. Korean J. Ecol. Environ..

[50] Li X., Dreher T.W., Li R. (2016). An overview of diversity, occurrence, genetics and toxin production of bloom-forming *Dolichospermum* (*Anabaena*) species. Harmful Algae.

[51] Li L., Wan N., Gan N.Q., Xia B.D., Song L.R. (2007). Annual dynamics and origins of the odorous compounds in the pilot experimental area of Lake Dianchi, China. Water Sci. Technol..

[52] Li Z., Hobson P., An W., Burch M.D., House J., Yang M. (2012). Earthy odor compounds production and loss in three cyanobacterial cultures. Water Res..

[53] Smucker N.J., Beaulieu J.J., Nietch C.T., Young J.L. (2021). Increasingly severe cyanobacterial blooms and deep water hypoxia coincide with warming water temperatures in reservoirs. Glob. Change Biol..

[54] Wan L., Chen X., Deng Q., Yang L., Li X., Zhang J., Song C., Zhou Y., Cao X. (2019). Phosphorus strategy in bloom-forming cyanobacteria (*Dolichospermum* and *Microcystis*) and its role in their succession. Harmful Algae.

[55] Nielsen A., Trolle D., Me W., Luo L., Han B.P., Liu Z., Olesen J.E., Jeppesen E. (2013). Assessing ways to combat eutrophication in a Chinese drinking water reservoir using SWAT. Mar. Freshw. Res..

[56] Espinosa C., Abril M., Guasch H., Pou N., Proia L., Ricart M., Ordeix M., Llenas L. (2020). Water flow and light availability influence on intracellular geosmin production in river biofilms. Front. Microbiol..

[57] Howard C.S. (2020). Taste and Odor Event Dynamics of a Midwestern Freshwater Reservoir. Ph.D. Thesis.

[58] Alghanmi H.A., Alkam F.A.M., Al-Taee M.M. (2018). Effect of light and temperature on new cyanobacteria producers for geosmin and 2-methylisoborneol. J. Appl. Phycol..

[59] Watson S.B., Ridal J. (2004). Periphyton: A primary source of widespread and severe taste and odour. Water Sci. Technol..

[60] Zhou B., Yuan R., Shi C., Yu L., Gu J., Zhang C. (2011). Biodegradation of geosmin in drinking water by novel bacteria isolated from biologically active carbon. J. Environ. Sci..

[61] Hoefel D., Ho L., Monis P.T., Newcombe G., Saint C.P. (2009). Biodegradation of geosmin by a novel Gram-negative bacterium; isolation, phylogenetic characterisation and degradation rate determination. Water Res..

[62] Durrer M., Zimmermann U., Jüttner F. (1999). Dissolved and particle-bound geosmin in a mesotrophic lake (Lake Zurich): Spatial and seasonal distribution and the effect of grazers. Water Res..

